# Use of Plasmid pVMG to Make Transcriptional ß-Glucuronidase Reporter Gene Fusions in the *Rhizobium* Genome for Monitoring the Expression of Rhizobial Genes In Vivo

**DOI:** 10.1186/s12575-019-0096-y

**Published:** 2019-05-03

**Authors:** Mengsheng Gao, Anne Benge, Tai-Jung Wu, Regina Javier

**Affiliations:** Soil and Water Sciences Department, Cancer and Genetics Research Complex, University of Florida-Institute of Food and Agricultural Sciences, Room 330E, Gainesville, 32610 USA

**Keywords:** Rhizobia symbiosis, ß-glucuronidase gene (*uidA)*, Transcriptional fusions, Chromosomal *uidA* transcriptional fusions

## Abstract

**Background:**

The soil bacterium *Sinorhizobium meliloti* and its allies are important nitrogen-fixing bacterial symbionts that cause N_2_-fixing nodules on the roots of legumes. Chromosomal ß-glucuronidase gene (*uidA)* transcriptional fusions are frequently used to monitor the expression of bacterial genes during the symbiosis. However, the construction of the fusions is laborious.

**Results:**

The narrow-host-range, fusion selective plasmid pVMG was constructed and used as a vector for the construction of chromosomal *uidA* transcriptional fusions in the *S. meliloti* genome. Translation termination codons were added in all three reading frames upstream of the promoterless *uidA* in this vector to ensure transcriptional fusions. pVMG replicated to high copy number in *Escherichia coli*, offering advantages for the isolation of fusion-containing plasmids and the restriction analysis. Genomic locations of *uidA* fusions were verified in a simple PCR experiment. All these helps reduce the sample processing time and efforts. As a demonstration of its usefulness, the N-acyl homoserine lactone (AHL) signal synthase gene promoter was fused to *uidA* and shown to be expressed by *S. meliloti* in the senescence zone of the nodule on the host plant, *M. truncatula*. This indicates the presence of AHL signals at the late stages of symbiosis.

**Conclusions:**

A simple, pVMG-based method for construction of chromosomal *uidA* transcriptional fusions has been successfully used in the model rhizobium *S. meliloti*. It is also applicable for other rhizobial strains.

**Electronic supplementary material:**

The online version of this article (10.1186/s12575-019-0096-y) contains supplementary material, which is available to authorized users.

## Background

The chromosomal *uidA* transcriptional fusions are frequently used for monitoring in vivo expression of bacterial genes for at least three reasons: First, the fusions allow the transcriptional activities of bacterial genes to be monitored at their nature levels [1–4]. Second, the fusions do not need antibiotics for maintaining their stability in the genome. Third, they avoid problems that associate with replicating plasmid systems which can disrupt regulation of expression due to copy number effects [1, 3–5]. As higher plants lack ß-glucuronidase activity [6], the *uidA* gene provides a sensitive enzyme assay for which a broad range of substances are available.

Methods for making chromosomal *uidA* transcriptional fusions in rhizobia involve a bacterial narrow-host-range, plasmid vector with a promoterless *uidA* gene [1, 2, 4]. Segments of DNA containing gene promoter from rhizobia can be cloned into the multiple cloning sites (MCS) located upstream of the *uidA* in the vector*.* The fusion-containing plasmids are maintained in a suitable *E. coli* strain, isolated and restriction analyzed, and then can be transferred to a rhizobial strain from *E. coli* in bi- and tri-parental mattings. As the vector uses an origin of replication (e. g., pUC) that is inactive in rhizobial strains [7], each fusion-containing plasmid co-integrates into the rhizobial host with the rhizobial host DNA. This can create a single copy *uidA* transcriptional fusion in the rhizobial host genome. The genomic locations of the fusions are typically verified by Southern blotting. The integration of fusion-containing plasmid does not disrupt the targeted locus if the cloned DNA fragment in the plasmid will not be internal to the transcription unit [1, 8].

A few plasmids have been used as narrow-host-range *uidA* transcriptional vectors in pioneering studies of rhizobial gene expression: pMH11 [9], pVO155 [1, 2], and pTH1522 [4]. While very fruitful to make *uidA* transcriptional fusions for in vivo studies, some of those vectors lack translational termination codons between their MCS and promoterless *uidA* reporter gene, therefore require additional experiments to ensure transcriptional fusions. Others are low copy number plasmids which can make restriction analysis and isolation ineffective. In some of those methods, Southern blotting was used to verify genomic locations of *uidA* fusions. While specific and sensitive, Southern blotting is time-consuming. Therefore, we have constructed a transcriptional *uidA* fusion selective plasmid pVMG on the basis of pVO155 [1] and developed a pVMG-based method for the construction of chromosomal *uidA* transcriptional fusions in the rhizobial genome. We showed that PCR has the necessary combination of simple procedure, sensitivity and consistent results thus can be used in the place for verifying *uidA* fusions. We demonstrated the utility of the pVMG and the pVMG-based method by constructing and testing a transcriptional fusion between the *sinI* gene of *S. meliloti* and *uidA*.

## Methods

### Bacterial Strains and Media

All strains and plasmids used are listed in Table 1. The following media were previously described: LB, a complex medium [13]; TY, a complex medium for *S. meliloti* [14], containing, Per liter, tryptone 6 g; yeast extract 3 g, and CaCl_2_^.^2H_2_O 0.5 g. Final concentrations of antibiotics: 100–200 μgml^− 1^ of neomycin (Nm) and 250–500 μgml^− 1^ of spectinomycin (Sm) (for the *S. meliloti* strains).

**Table 1:** Strains and plasmids

Strain or plasmid	Relevant features	Source or Reference
Strains		
DH5a	a-complementation	Invitrogen
8530	*S. meliloti*, *expR*+, *sinI*+, Sm^r^	[10]
1021	*S. meliloti*, *expR*-, sinI+, Sm^r^	[11]
MG32	8530 with *sinI* deletion *expR*+, *sinI*^−^, Sm^r^	[12]
8530 *sinI*::VMG495	*sinI-uidA* transcriptional fusion, *expR*+, *sinI*+, Sm^r^, Nm^r^	This work
1021 *sinI*::VMG495	*sinI-uidA* transcriptional fusion, *expR*+, *sinI*+, Sm, Nm	This work
MG32 *sinI*::VMG495	*sinI-uidA* transcriptional fusion, *expR*+, *sinI-*, Sm^r^, Nm^r^	This work
8530 nop::VMG209	Non promoter (nop) DNA-*uidA*, *expR*+, *sinI*+, Sm^r^, Nm^r^	This work
Plasmids		
pVO155	pUC19-derived integrational *uidA* vector	[1]
pVMG	pVO155 with stop codons upstream of promoterless *uidA* in all ORFs	This work, [12]
pRK600	pRK2013 Nm::Tn9, Cm^r^	[1]
pVMG495	pVMG, *sinI* 5’-end, transcriptional fusion	This work
pVMG209	pVMG, nop DNA of *S. meliloti*.	This work

### Biochemistry

#### DNA Biochemistry

Restriction endonucleases were purchased from New England Biolabs (New England Biolabs, Inc. Beverly, MA, USA) and used according to the manufacturer’s instructions. A 25-bp linker was created by annealing two complementary oligonucleotides (5′- GATCCCTCGAGCTGACTAACTAGCT-3’; 5’-CTAGAGCTAGTTAGTCAGCTCGAGG-3′). The linker contained a *Xho* I site and translation termination codons in three different ORFs with termini cohesive to either *Bam*H I or *Xba* I. DNA ligations were performed with T4 DNA ligase (Biolabs, # M0202 T). Colony PCR was performed in a sterile 0.5-ml amplification tube containing 1 x Standard *Taq* Reaction Buffer (Biolabs #B9014S), 0.5 μM of four dNTPs, 0.2 μM of two forward and revers primers, 1 unit of *Taq* DNA polymerase (Biolabs #M0320 L) and an individual *S. meliloti* colony. PCR primers are shown in Table 3. The nucleic acids were amplified for 35 cycles. The denaturation, annealing, and polymerization times and temperatures were 1 min at 94 °C, 1 min at 50 °C, and 30 s. at 72 °C. DNA sequencing was performed by Rightmire DNA Sequencing Facility at The Ohio State University, Columbus, Ohio, and by the DNA Sequencing Laboratory at the Interdisciplinary Center for Biotechnology Research at University of Florida, Gainesville, Florida.

Genomic DNA extraction was described previously [4] with minor modifications. DNA was prepared from 5 ml of culture grown in TY broth with appropriate antibiotics to saturation with shaking. Bacteria were collected in 2-ml microcentrifuge tubes by centrifugation, washed once with 0.85% NaCl, and then resuspended in 750 μl of TE buffer (10 mM Tris-HCl, pH 8.0, mM EDTA). Sodium dodecyl sulfate (SDS) was added to 1%, NaCl to 1 M, and proteinase K (Sigma) to 0.5 mg/ml. Samples were mixed gently and incubated at 65 °C for 2 h for complete lysis. DNA was extracted twice with equal volume of buffer-saturated phenol, twice with 1:1 phenol-chloroform, and once with chloroform, and then NaCl was added to 0.2 M and nucleic acids precipitated with a 2.5 volumes of ethanol. The pellet was dissolved in 400 μl of TE with 20 g/ml RNase A and incubated for 30 min at 37 °C. DNA was extracted once with 1:1 phenol-chloroform and once with chloroform, ammonium acetate was added to 0.5 M, and DNA was precipitated with an equal volume of isopropanol. The optical density at 260 nm (OD_260_) of the sample was measured to determine the DNA concentration. The samples were then lyophilized and dissolved to proper concentrations for restriction enzyme digestions.

#### Acyl Homoserine Lactones (AHL) Biochemistry

C_16:1_-Δ^9^*cis*-(L)-homoserine lactone (referred to as “C_16:1_-HSL” in text) was from Cayman Chemical (Ellsworth Road, Ann Arbor, MI U.S.A.). The molecule was dissolved in ethyl acetate at stock and diluted in methanol. AHL was added in a liquid medium prior to inoculation of bacteria.

#### ß-Glucuronidase (GUS) Biochemistry

Quantitative assay for GUS activity was described previously [6] with modification [15]. Cells were permeabilized with lysozyme (200 μg ml^− 1^, 37 °C for 10 min), and the GUS activity was measured with PNPG (p-nitrophenyl-β-*o*-glucuronide). GUS activity was calculated in nanomoles per minute per OD_595_ unit × 1000 as in [15]). Color producing substrate X-gluc (5-brome-4-chloro-3-indolyl-β-D-glucuronide) for GUS enzyme to act was used for visualizing activity of GUS in histochemical assay at working concentration of 40 μg/ml as described before [16].

### Conjugations

Recombinant plasmids were maintained in *E. coli* DH5α and were conjugated into *S. meliloti* recipient strains with help plasmid pRK 600 by bacterial conjugation method [1] with modifications. Log-phase recipient cells (10^+ 8^/ml) were used in the conjugation. The donor and recipient ratio was approx. 8:1*. S. meliloti* transconjugants were selected on TY agar medium at the present of neomycin.

### Sequence Analysis

Sequence assembly was performed with MacVector with Assembler 12.01. Database searches were conducted through the *S. meliloti* genome web page using blastn.

### Plant Growth and Nodulation

Cultivation of *M. truncatula* A17, root nodulation, and nodule harvesting were described previously [17].

## Results

### Construction of pVMG

The narrow-host-range plasmid pVO155, with a MCS, a promoterless *uidA* (*gus*) reporter gene encoding ß-glucuronidase (GUS), and a pUC origin of replication, was used as a base for the construction of pVMG.

**Fig. 1:**
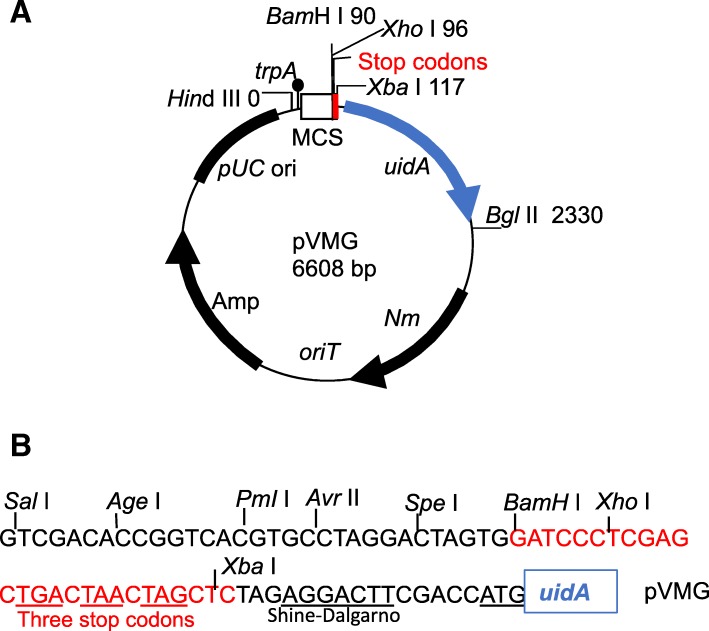
Structure and multiple cloning sites (MCS) of the plasmid pVMG. **a**. pVMG, a derivative of pVO155, contains a short DNA linker (red) for translation termination codons to ensure transcriptional fusions. The size of pVMG was deduced from the known sequence of the analogous plasmid pVMGTnpR (GenBank accession number EU232661). **b**. The MCS of pVMG. All the restriction sites are usable except the *Xba* I site. The *Xba* I is located after translation termination codons, therefore not readily usable for making transcriptional fusions

pVMG was constructed by replacing a *Bam*H I-*Xba* I fragment at the end of MCS in pVO155 with the 25-bp *Bam*H I-*Xba* I synthetic DNA linker containing three translation terminations and a *Xho* I site (see Methods). The structure of pVMG is shown in Fig. 1a. The translation termination codons (referred to as to “stop codons” in Figure) upstream of the *uidA* in all reading frames ensure transcriptional fusions (Fig. 1b). DNA sequencing and double/triple endonuclease digests of the plasmid confirmed that pVMG retains the original promoterless *uidA* gene, seven of the 8 restriction enzymes in multiple cloning sites (MCS), a *E. coli trpA* terminator upstream of the MCS to prevent read-through from the vector, a unique *Hin*d III site adjacent to the *trpA*, a unique *Bgl* II and the pUC origin of replication. DNA sequencing data also confirmed that pVMG retains the original *oriT* origin of transfer, the neomycin (Nm) resistance ORF and the ampicillin (Amp) resistance ORF.

pVMG replicates to high copy number in *E. coli* DH5a. This provides cloning advantages. The average DNA yield of pVMG in Miniprep experiments was similar to the yield of pUC19 itself (Table 2). Various 200–800 bp DNA fragments from *S. meliloti* were cloned into pVMG [12, 18, 19]. For an insert size of 200–800 bp, we typically obtained 80–190 Nm^R^ colonies by using the 1/5 volume of each ligation reaction and of these, approximately 85% contained the expected inserts.

**Table 2:** DNA yields of pVMG, pVO155 and pUC19 grown in medium LB

Plasmid	Average yield *n* = exp. (Μg)
pUC19	18 ± 3 (*n* = 5)
pVMG	16 ± 2 (*n* = 75)
pVO155	16 ± 2 (*n* = 17)

QIAprep Spin Miniprep Kit was used to purify DNA from 1.5 ml LB overnight cultures of DH5a containing pUC19, or pVMG or pVO155. Elution was performed according to the standard protocol (50 μl Buffer EB and 1 min incubation). Use of the recommended LB composition (with 10 g/liter NaCl) and Nm resistance provides optimal plasmid yield. Time spent was < 15 min. Costs per μg DNA was 7 cents.

### Construction of Transcriptional *sinI-uidA* Fusions

**Fig. 2:**
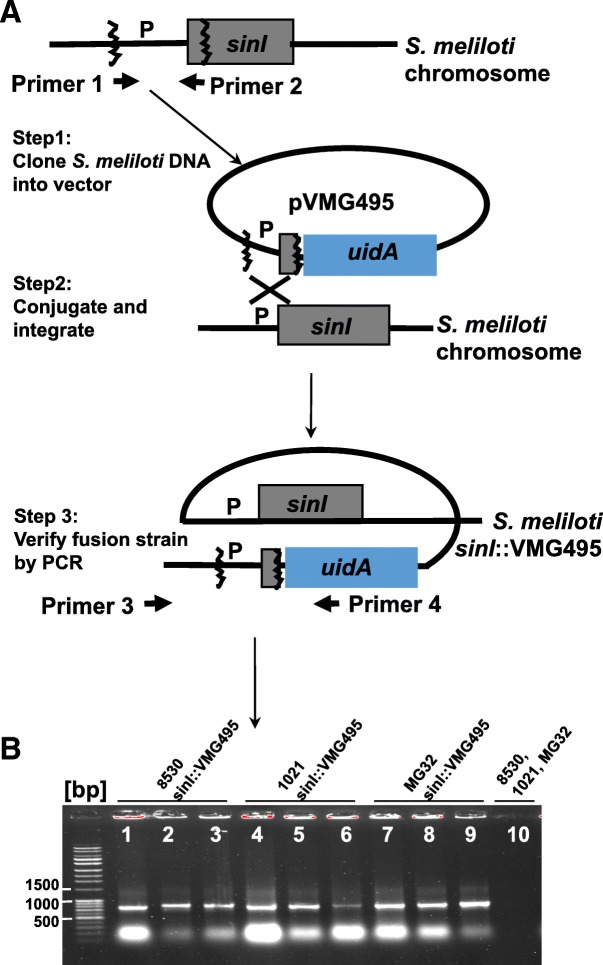
Testing the pVMG method. **a**. pVMG method overview. **b**. Gel electrophoresis of PCR-amplified 720-bp DNA. 720-bp PCR-bands were specific for *S. meliloti* reporter fusion strains 8530 *sinI*::VMG495, MG32 *sinI*::VMG495, and 1021 *sinI::*VMG495. DNA marker: The Invitrogen TrackIt 1 Kb Pluse DNA Ladder

In order to demonstrate the usefulness of pVMG, we made a transcriptional *sinI* gene reporter fusion to *uidA* by using a pVMG-based method (Fig. 2a). The *sinI* gene of *S. meliloti* encodes the synthase of the bacterial AHL signaling molecules [20]. The *sinI* gene mutations abolish the transcription of *sinI*-dependent genes and delay initiation of nodulation on the roots of the host plant, *M. truncatula* [12]. The *sinI* promoter is inducible by C_16:1_-HSL and the ExpR protein of *S. meliloti* enhances this induction [10]. The *sinI* gene is expressed in free-living bacteria and at the time when cells invade the nodule of *M. truncatula* [12]. In our study of the *Rhizobium sinI* gene function, we wished to examine the expression of *sinI* gene in late stages of the symbiosis.

The intact 5’-end of *sinI* gene fragment was cloned into pVMG. This 438-bp fragment contains the *sinI* promoter and ExpR binding site (nucleotides − 416 to + 22 with respect to the *sinI* translation start site) [21]. The fragment was amplified from chromosome of *S. meliloti*. The oligonucleotide primers (Primer 1-*sinI* and Primer 2-sinI) used for the amplification are shown in Table 3. The Primer 1 contains a *Sal* I site and the primer 2 contains a *Bam*H I site. The PCR-amplified fragment was treated with *Sal* I and *Bam*H I, then ligated to the *Sal* I-*Bam*H I digested pVMG. This ligation created a transcriptional *sinI-uidA* fusion-containing plasmid pVMG495. pVMG495 was transferred into Invitrogen DH5a competent cells which are neomycin sensitive (Nm^S^).

**Table 3:** Primers used in the study. *Sal* I and *Bam*H I restriction sites are underlined.

Name	Sequence (5′-3′)	usage
Primer 1-*sinI*	ACGCGTCGACGTTGAGTGGTCCGCCTACCG	*sinI* forward
Primer 2-*sinI*	GCTGCGACCGGATCCGTTCACTAT	*sinI* reverse
Primer 3-*sinI*	GGTGGAATGGGCGACAGCGCG	*sinI* forward
Primer 4	GGGTTGGGGTTTCTACAGGA	*uidA* reverse
Primer 1-nop	AGCCTTGAACGTCGACTG	nop forward
Primer 2-nop	ATGGAGGATCCAGCGAG	nop reverse
Primer 3-nop	AAGTTGGGGTATCGCCCTAAA	nop forward

pVMG495 was conjugated into the *S. meliloti expR* mutant strain 1021 [11] by a tri-parental matting with pRK600 as a helper. *S. meliloti* conjugants were selected for neomycin resistance (Nm^R^), yielding a transcriptional *sinI-uidA* fusion strain called *S. meliloti* 1021 *sinI*::VMG495 (Table 1).

In the experiments reported above, the co-integration frequency of the plasmid pVMG with the 438-bp chromosomal insert in the *S. mlilloti* 1021 was about 4.3 X 10 ^− 5^ (number of Nm^r^ recombinants per total number of receipted cell present). This represents about a 8.6-fold increase over the co-integration frequency obtained in *Azorhizobium caulinodans* ORS571 [22] for a pBBR-based replicon with a larger insert introduced by a similar tri-parental matting method. In a control experiment, the vector pVMG was conjugated into the strain 1021, no Nm^r^- transformants were obtained.

Two additional transcriptional *sinI-uidA* fusion strains were constructed by conjugating pVMG495 into *S. meliloti sinI* mutant strain MG32 [12] and *S. meliloti* wild-type strain 8530 [10], respectively. They yielded *S. meliloti* MG32 *sinI*::VMG495 and *S. meliloti* 8530 *sinI*::VMG495 (Table 1).

All *S. meliloti sinI-uidA* fusion strains (1021 *sinI*::VMG495, MG32 *sinI*::VMG495, 8530 *sinI*::VMG495) were verified for the site-specific integration of pVMG495 by PCR. Three colonies of each fusion strain candidates were analyzed. The oligonucleotide primers (Primer 3-*sinI* and Primer 4) used for the amplification are shown in Table 3. The integration specific Primer 3-*sinI* attaches the genomic DNA region outside and upstream of the cloned 438-bp fragment. The *uidA* specific primer 4 attaches DNA region inside of the *uidA* from pVMG and it faces toward the cloned *sinI* fragment (Fig. 2a). Specific primers amplified the ~ 720-bp DNA region of integration (Fig. 2b, lanes 1–9) from all tested fusion strains, but not from their parental strains (Fig. 2b, lane 10). The fusion-strain-specific amplification strongly suggests the site-specific integration of pVMG495. The 720-bp product was presumably constituted of the cloned *sinI* fragment flanked by the upstream *S. meliloti* chromosome and the 5-end of *uidA* gene. The identity of the 720-bp product was determined by DNA sequencing. This confirmed the presence of all expected DNA segments as well as a termination codon in-frame with the *sinI* gene (see Additional file 1).

Next, we checked the identity of *S. meliloti* reporter strains (1021 *sinI*::VMG495, MG32 *sinI*::VMG495 and 8530 *sinI*::VMG495) by the method for recovery of integrated plasmid for subsequent DNA sequencing [1, 8]. We cut the entire genome with a restriction enzyme that does not cut within the pVMG495 plasmid. We then circularized the fragments with T_4_ DNA ligase and transformed them into *E. coli* strain DH5a. We recovered a plasmid that contains the expected structure in every strain.

Subsequently, we checked the identity of *S. meliloti* reporter strains (1021 *sinI*::VMG495, MG32 *sinI*::VMG495 and 8530 *sinI*::VMG495) by Southern blot [23]. As shown in Additional file 2 Figure S2 , Southern blot test detected one fragment of *Hin*d III digestion in the DNA from each sample of the fusion strains of *sinI*::VMG495. The identical band was visible at approx. 7.7-kb from each fusion sample since the *sinI* gene integration regions are identical. This band was caused by the chimeric DNA fragment of genome-pVMG495. The Southern blotting test detected no additional second or third copies of the vector in the genomic DNA samples, suggesting no random integrated vector DNA in the genomes of 1021 *sinI*::VMG495, MG32 *sinI*::VMG495 and 8530 *sinI*::VMG495.

By using the pVMG method shown in Fig. 2a, we constructed a control strain called *S. meliloti* 8530 nop::VMG209 (Table 1) for measuring background activity of the GUS. In this experiment, a plasmid called pVMG209 was created by cloning a 209-bp non-promoter (nop) DNA segment of *S. meliloti* to the *Sal*I-*Bam*HI site of pVMG. pVMG209 was integrated into the chromosome of the *S. meliloti* 8530 strain. The primers we used for the construction of 8530 nop::VMG209 strain are shown in Table 3.

### Testing Transcriptional *sinI-uid*A Fusions

The fusion strains and the control strain were tested for responsive changes in GUS activity in free-living bacteria. The rhizobium strains were cultured in (a) TY broth, (b) TY broth containing 7.5 nM of C_16:1_-HSL as we did before [24]. The fusion in *sinI* promoter was found to have significant changes in GUS activity in response to *expR sinI*, *sinI*, *expR* backgrounds or to added AHL when tested at late log phase (optical density at 600 nm [OD_600_] = 0.98 to 1.08.) (Table 4). Quantitative GUS assay demonstrated that the *sinI* promoter activity in the presence of C_16:1_ was 5.8-fold higher than in the absence of C_16:1._ This induction factor is consistent with the one measured in a *S. meliloti* reporter strain carrying a chromosomal single copy of *sinI-lacZ* transcriptional fusion [25]. The *uidA* fusion in non-promoter (nop) DNA had no activity in WT background (Table 4) and was not responsive to the AHL (data not shown).

**Table 4:** Responses of single copy *sinI-uidA* transcriptional fusion to *expR sinI*, *sinI*, *expR*, and AHL in medium TY

Strain	Genotype	ß-glucuronidase activity^a,b^
8530 *sinI*::VMG495	*expR+, sinI+*	194 ± 4
MG32 *sinI*::VMG495	*expR+, sinI-*	33 ± 3
MG32 *sinI*::VMG495 (C_16:1_*)*	*expR+, sinI-*	192 ± 2
1021 *sinI*::VMG495	*expR-, sinI+*	95 ± 3
8530 nop::VMG209	*expR+, sinI+*	< 0.1

^a^Nanomoles per minute per OD_595_ [15].

^b^Shown are averages and standard deviations (*n* = 3).

The wild-type strain that expresses ß-glucuronidase (GUS) was also tested for its ability to initiate nodulation on roots of *M. truncatula* plants, using a previously described protocol [17]. The rate and the efficiency of nodule initiation by the GUS expressing strain 8530 *sinI*::VMG495 and the 8530 parent were found similar. This observation indicated that 8530 *sinI*::VMG495 was normal in the ability to initiate nodulation on the host plant*.* The normal initiation of nodulation suggested that *sinI* activity was not inhibited by the GUS or by the *sinI* fragment of 8530 *sinI*::VMG495 strain.

**Fig. 3:**
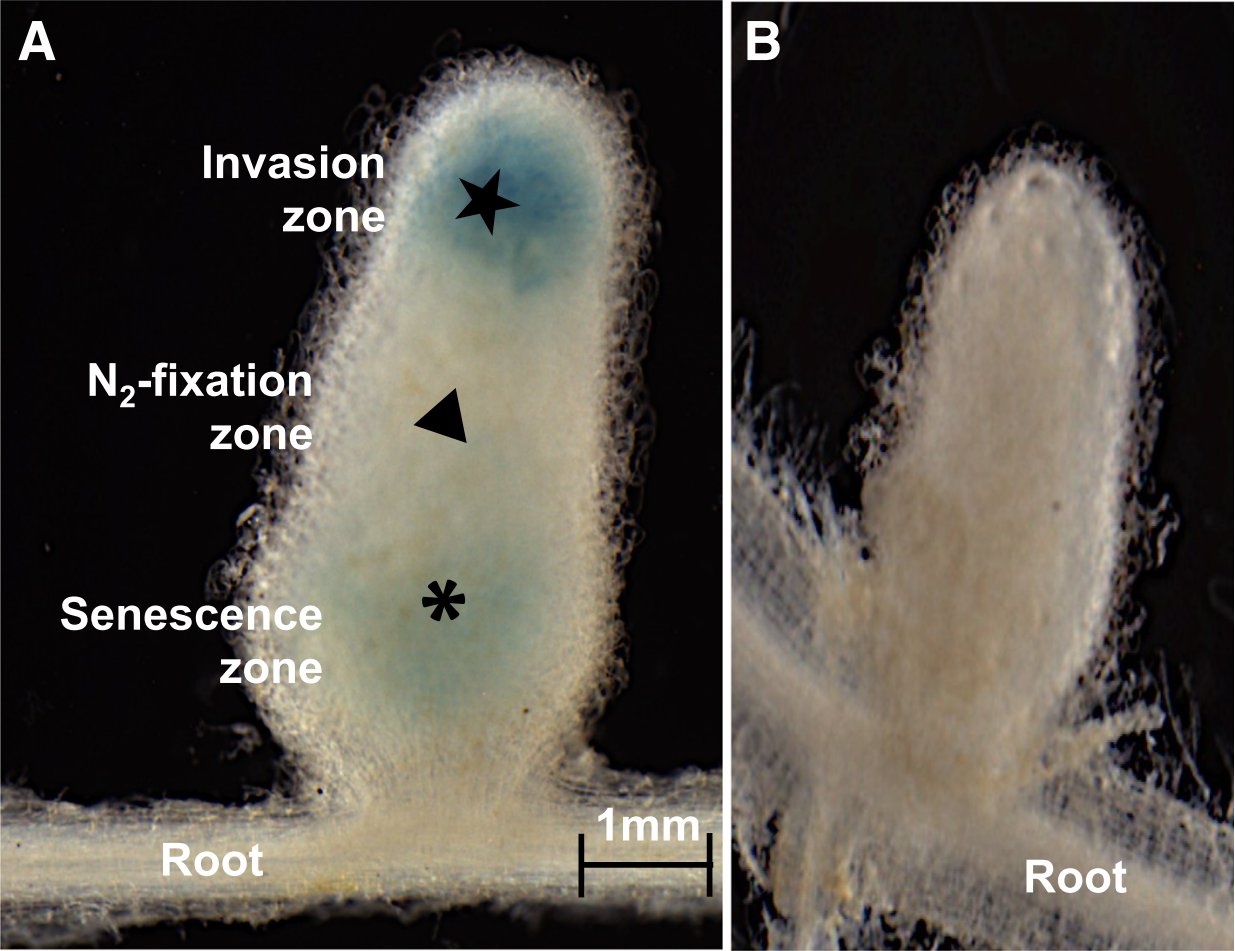
Histochemical assay of *M. truncatula* nodules. **a** 8530 *sinI*::VMG495 (*n* = 13) **b** 8530 nop::VMG209 (*n* = 5). Nodules were stained with 4 mg /ml X-Glue in NaH_2_PO4. pH 7.2, 37 °C as described [26] for 3 h (**a**) or for 48 h (**b**). Photographs were taken 7 weeks after inoculation with Olympus MVX10 dissecting scope equipped with a MicroFire camera (Optitronics, Goleta, CA, USA)

In order to examine the expression of *sinI* gene in the late stages of the symbiosis, roots of *M. truncatula* were inoculated with 8530 *sinI*::VMG495 and 8530 nop::VMG209. Nodules were harvested and stained with X-Gluc [6]. Stained nodules were examined under a dissecting scope. The 8530 *sinI*::VMG495 infected nodules showed a blue invasion zone and a blue senescence zone (Fig. 3a). The 8530 nop::VMG209 infected nodules did not (Fig. 3b). While confirming the expression of the *sinI* by *S. meliloti* bacteria at the time of nodule invasion [27], the results demonstrated the expression of the *sinI* in the senescence zone of the *M. truncatula* root nodule. This indicates the presence of AHL signals at the late stages of symbiosis.

## Discussion

Several reporter gene systems are available to monitor bacterial gene activity within plant cells. These include *ß-galactosidase* (*lacZ*) [28], *green fluorescent protein* (*gfp*) [29] and *ß-glucuronidase* (*uidA*) [6]. *uidA* reporter gene system has increased sensitivity relative to *lacZ* because most higher plants show large amounts of galactosidase activity but they lack ß-glucuronidase (GUS) activity [6]. *uidA* reporter system has increased sensitivity relative to GFP when used within root nodule cells because molecular oxygen (O_2)_ is low in those cells but GFP strictly requires O_2_ for maturation of fluorescence [30]. Because it has increased sensitivity, *uidA* has been extensively used to monitor bacterial gene activity during the *Rhizobium*-legume symbiosis [1, 2, 4, 19, 31, 32]*.*

In the course of research in *S. meliloti*, we have developed a new transcriptional *uidA* fusion selective vector that preserves properties of its parent plasmid pVO155, i.e., to be a mobilizable narrow-host plasmid and present at a high copy number in *E. coil*. The characteristic presence of translation terminations between the MCS and the promoterless *uidA* gene in a pUC-based replicon, distinguishes pVMG as an effective vector to make chromosomal *uidA* transcriptional fusions in Rhizobia. This effectiveness of making transcriptional fusion, together with the simple procedure of verifying fusion by PCR, distinguish pVMG method as an effective method for various applications in construction gene fusions of Rhizobium. We have found that Rhizobium DNA fragments with size up to 3.5-kb can be easily cloned in pVMG [12, 18, 19] and yields of those Rhizobium fusion-containing plasmids from minipreps were similar to the yield of pUC19 which is much higher than those of pBBR-based narrow-host plasmids. Large amount of candidate rhizobial *uidA* fusion strains can be verified easily by a simple one-step colony PCR which is much easier than Southern blotting or recovery of integrated plasmid for verification of fusion. Additionally, a PCR product could be used to identify the genomic site of integrated plasmid by direct DNA sequencing.

Genomic sites of integrated plasmid have been typically verified with the Southern blotting procedure [23]. Southern blotting is extremely sensitive and specific for detecting DNA in a particular sample and has been used in diagnostic studies to detect genomic sites of integrated plasmid in bacteria [2, 12, 22, 33, 34]. Although sensitive and specific, Southern blotting requires isolation of DNA, digestion of DNA with restriction enzyme (s), separation of DNA by agarose gel electrophoresis, blotting and hybridization with a probe [16]. Making a probe for hybridization involves generating, purifying and labeling DNA. In most cases, radioactive probes are used [16]. While generally effective, these methods are time consuming and create the potential for radiation exposure.

In an effort to reduce sample processing time and efforts, we have verified the genomic site of the integrated plasmid by the simple PCR experiment for subsequent DNA sequencing. Results from this verification method were checked with other two conventional verification methods: 1) the method for recovery of integrated plasmid for genome-vector site identification [8, 35] and 2) Southern blotting [23]. Results from both conventional verification methods supported the conclusion that the PCR verification method has the necessary combination of simple procedure, sensitivity and specificity. Thus, the introduction of the PCR verification method can reduce the use of Southern blotting in diagnostic studies for verification of genomic sites of integrated plasmid.

As shown in Fig. 2a, the genome of *S. meliloti uidA* reporter strain contains the vector DNA. Because the vector DNA has not affected any known genes for the symbiosis and does not appear to affect the growth of the bacteria, the symbiotic behaviors of the reporter strain are as normal as its parental strain. When one wishes to use pVMG to make a chromosomal *uidA* fusion while avoiding the vector DNA, one possibility becomes apparent. It is possible to use *tnpR* encoded resolvase [36] to excise the vector DNA from the genome. The resolvase catalyzes excision of the DNA flanked by short DNAs called res sequences. pVMG (Fig. 1a) has a unique *Hin*dH III site upstream of the MCS and a unique *Bgl* II site downstream of *uidA*, allowing insertion of the res sequences for the resolvase to function. Similar types of work have been successfully carried out in *S. meliloti* for other target region excision purposes and a version of pVMG carrying a inducible *tnpR* exists [3]. Other techniques might be of use. In CRISPR-Cas9, for example, the class of RNA-guided endonucleases known as Cas9 from the microbial adaptive immune system CRISPR (clustered regularly interspaced short palindromic repeats) can be targeted to virtually any genomic location of choice by a short RNA guide [37]. With two such guides, a CRISPR-Cas9 system has generated target excisions in the genomes of bacteria *Streptococcus pneumoniae* and *E. coli* [38]. Given the results of work with those bacteria, CRISPR-Cas9 might be of use to excise the vector DNA from the genome of *S. meliloti.*

In addition to the generation of transcriptional *uidA* fusions in a DNA region from *S. meliloti*, we have been using pVMG for generating genome-wide transcriptional *uidA* fusions in the bacterium. We have cloned a library of the *S. meliloti* DNA fragments to the *Bam*HI site of pVMG and screened the resulting strains for the *sinI*-regulated activity of transcription in the late stages of symbiosis. Preliminary studies of transcription of *S. meliloti* genes linked expression of some of these genes to the activity of the *sinI* gene (unpublished results). In fact, the analogous plasmid pVO196 [1] has already been successfully used for generating a library of *S. meliloti* transcriptional fusions to a promoterless copy of *bacA* gene for discovering activity of the rhizobial genes in the intermediate stages of the symbiosis. According to our preliminary studies and the published study, it is likely that pVMG will be useful for the *in planta* bacterial transcriptome studies*.*

## Conclusions

A simple method has been developed for making chromosomal *uidA* transcriptional reporter fusions in *S. meliloti*. The method is based on the narrow-host-range, high copy number, transcriptional *uidA* fusion selective pVMG for the effectiveness of the fusion construction. The fusions are verified by a simple colony PCR and a PCR product could be used to identify the fusion site by direct DNA sequencing. The method is successfully used in the model rhizobia *S. meliloti.* The method is also applicable to many other rhizobia stains, but it is not applicable to those that are resistant to both neomycin and ampicillin.

## Additional Files

Additional File 1: The DNA sequence of the 720-bp PCR product. (DOCX 3534 kb)

Additional File 2: Southern blot of DNAs of *S. meliloti* strains. (DOCX 5227 kb)
