# Supplementary material for: Use of Plasmid pVMG to Make Transcriptional ß-Glucuronidase Reporter Gene Fusions in the Rhizobium Genome for Monitoring the Expression of Rhizobial Genes In Vivo
Source: Biol Proced Online. 2019 May 3;21:8. doi: 10.1186/s12575-019-0096-y (PMC6498626; doi:10.1186/s12575-019-0096-y)
Supplement: Supplementary file 1 — The DNA sequence of the 720-bp PCR product. (DOCX 3534 kb) [file 12575_2019_96_MOESM1_ESM.docx]

**The DNA sequence of the 720-bp PCR product**
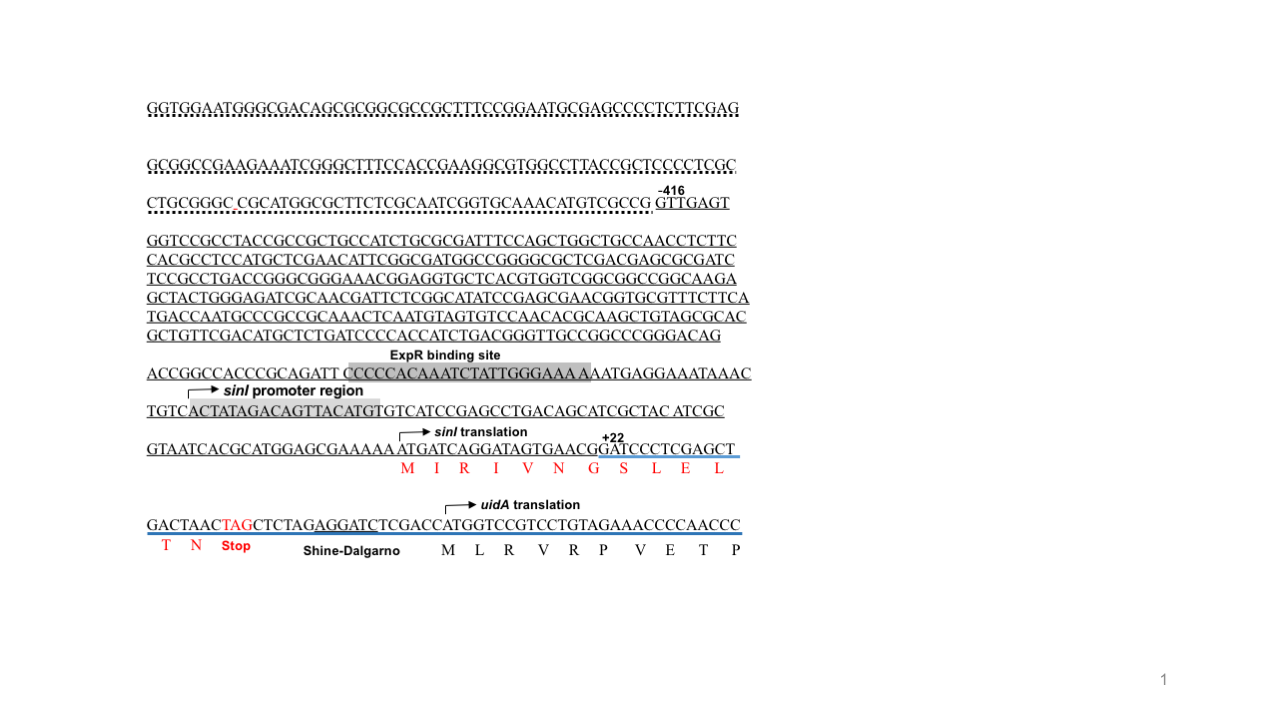


The dotted line indicates the region of *S. meliloti* chromosome upstream of the cloned 438-bp fragment. The solid line indicates the cloned 438-bp fragment itself (from -416 to +22). The blue line indicates the 5-end of *uidA* reporter gene in pVMG. Dark grey presents the ExpR binding site, while light grey presents the promoter region of the *sinI* gene. The stop codon sequence (red) downstream of the initiation codon of the *sinI* gene terminated the translation of the *sinI* and ensured a chromosomal single copy of the transcription fusion between the *sinI* gene and the promoterless *uidA* gene.
