# Supplementary material for: Use of Plasmid pVMG to Make Transcriptional ß-Glucuronidase Reporter Gene Fusions in the Rhizobium Genome for Monitoring the Expression of Rhizobial Genes In Vivo
Source: Biol Proced Online. 2019 May 3;21:8. doi: 10.1186/s12575-019-0096-y (PMC6498626; doi:10.1186/s12575-019-0096-y)
Supplement: Supplementary file 2 — Southern blot of DNAs of S. meliloti strains. (DOCX 5227 kb) [file 12575_2019_96_MOESM2_ESM.docx]

**Southern blot of DNAs of *S. meliloti* strains**

DNA was extracted from all test samples and each sample was digested with the restriction enzyme *Hin*d III. When the *sinI* gene was discovered (*Science* 2001, 293 (5530): 668-672), the gene region was examined carefully and found to lie in a region of the chromosomal DNA flanked by *Hin*d III restriction sites. The gene region lay in a fragment 9394 bp long. If the *sinI* is fused to pVMG495 at the expected site then the size of a resulting chimeric DNA fragment is about 7700-bp because of a *Hin*d III site in the integrated vector (see **Figure 1A**). If pVMG495 integrated at unexpected site, the illegitimately integrated vector DNA can also be determined by Southern blotting. The digested DNA was separated on an 8% agarose gel and transferred to nylon membrane, positively charged (Roche) for hybridization, the probe used in the test was a small piece of DNA from the vector and adjacent to the *Hin*d III site. This probe should detect one fragment of *Hin*d III digestion in the DNA from each sample of the fusion strains of *sinI*::VMG495, and this probe should detect no fragment from non-fusion chromosome. If a fragment with unexpected size or more than one fragments are detected in the material from any samples of fusion strains then this indicates that illegitimately integrations had occurred. **Additional Figure S2** shows how integration locations were detected using the Southern technique.

**Additional Figure S2**. Southern blot of DNAs of *S. meliloti* strains. (A) DNA samples on a 0.8 % agarose gel. (B) This photograph shows results of a Southern blot of the gel shown in panel A and is used to verify *sinI*::VMG495 fusion strains. Lane 1 contains DNA fragments of known molecular weight (Biolabs 2-log Ladder). Lane 2 shows DNA (the 1.9-kb templet DNA fragment for the vector probe) from the pVMG plasmid. Lane 3–5 show DNA from fusion strains 8530 *sinI*::VMG495, 1021 *sinI*::VMG495, and MG32 *sinI*::VMG495. Lane 6 and 7 show DNA from parental strains 8530 or MG32 and 1021. The DNA (from 3–7) was digested with restriction enzyme *Hin*d III, resolved on a 8 g agarose/l gel, blotted and probed with the vector probe. One identical band is visible at approx.7.7-kb (arrows) from each fusion sample since the *sinI* gene integration regions are identical. This band is caused by the chimeric DNA fragment (677-bp chromosomal DNA in addition to the 438-bp cloned *sinI* fragment and the 6600-bp fragment of pVMG vector). Only one band is visible since there is only one integration had occurred in each fusion strain. No bands were visible from any parental strains 8530/ MG32 and 1021. This is because the parental strains are non-fusion strains and do not contain the vector. (Lanes 1 and 2 were subject to the same hybridization but in a separate box. Lanes 6 and 7 were subjected to an extended exposure time).

In this experiment, Alkaline transfer of DNA on 0.8% agarose gels to Nylon Membranes (Roche) was done as described in Maniatis et al. (*Molecular Cloning: A Laboratory Manual* 1982, Cold Spring Harbor Laboratory, Cold Spring Harbor, NY). The vector probe (3 µg of template DNA) was labeled with DIG (digoxigenin-dUTP) (Roche Diagnostics GmbH, Roche Applied Science, Penzberg Germany) by using the Random Primed Labeling kit of Roche Applied Science. DNA was fixed on the filters. The filters were hybridized, washed and immunologically detected as instructed by an Instruction Manual of Roche.
